# A non-destructive approach for measuring rice panicle-level photosynthetic responses using 3D-image reconstruction

**DOI:** 10.1186/s13007-022-00959-y

**Published:** 2022-11-28

**Authors:** Jaspinder Singh Dharni, Balpreet Kaur Dhatt, Puneet Paul, Tian Gao, Tala Awada, Harel Bacher, Zvi Peleg, Paul Staswick, Jason Hupp, Hongfeng Yu, Harkamal Walia

**Affiliations:** 1 Department of Agronomy and Horticulture, University of Nebraska-Lincoln, Lincoln, NE 68583 USA; 2 Department of Computer Science and Engineering, University of Nebraska-Lincoln, Lincoln, NE 68588 USA; 3 School of Natural Resources, University of Nebraska-Lincoln, Lincoln, NE 68583 USA; 4 The Robert H. Smith Institute of Plant Sciences and Genetics in Agriculture, The Hebrew University of Jerusalem, Rehovot, Israel; 5 LI-COR Inc., 4647 Superior Street, Lincoln, NE 68505 USA

**Keywords:** Rice, Grain yield, Panicle, Carbon assimilation, Imaging, Heat stress, Photosynthesis, LiCOR 6800

## Abstract

**Background:**

Our understanding of the physiological responses of rice inflorescence (panicle) to environmental stresses is limited by the challenge of accurately determining panicle photosynthetic parameters and their impact on grain yield. This is primarily due to the lack of a suitable gas exchange methodology for panicles and non-destructive methods to accurately determine panicle surface area.

**Results:**

To address these challenges, we have developed a custom panicle gas exchange cylinder compatible with the LiCor 6800 Infra-red Gas Analyzer. Accurate surface area measurements were determined using 3D panicle imaging to normalize the panicle-level photosynthetic measurements. We observed differential responses in both panicle and flag leaf for two temperate Japonica rice genotypes (accessions TEJ-1 and TEJ-2) exposed to heat stress during early grain filling. There was a notable divergence in the relative photosynthetic contribution of flag leaf and panicles for the heat-tolerant genotype (TEJ-2) compared to the sensitive genotype (TEJ-1).

**Conclusion:**

The novelty of this method is the non-destructive and accurate determination of panicle area and photosynthetic parameters, enabling researchers to monitor temporal changes in panicle physiology during the reproductive development. The method is useful for panicle-level measurements under diverse environmental stresses and is sensitive enough to evaluate genotypic variation for panicle physiology and architecture in cereals with compact inflorescences.

**Supplementary Information:**

The online version contains supplementary material available at 10.1186/s13007-022-00959-y.

## Background

Rice (*Oryza sativa*) is crucial for global food security. However, rice production is susceptible to heat stress (HS) [1–7]. Rice reproductive development is considered the most heat-sensitive stage [8–12]. Even a short duration of heat stress during early grain development affects mature grain size and weight parameters [13–17]. During the reproductive stage, rice grain is the primary sink organ whose normal development depends upon the accumulation and utilization of photoassimilates from leaves [18, 19]. Recent studies suggest that in addition to being a temporary sink, panicles also contribute to the grain photoassimilate pool and consequently to grain yield [20–23].

A better understanding of source-sink dynamics in the context of photosynthetic responses and grain filling is needed for predicting how grain yield parameters are affected by temperature [24–27]. In absence of further improvement in rice heat resilience, it is estimated that for every 1 ℃ increase in temperature, there will be a ~ 3.2% decline in yield [28]. From a mechanistic perspective much of that impact could be due to the temperature sensitivity of the plant’s photosynthetic capacity and the cellular processes of developing seeds. Heat stress impacts photosynthesis in multiple ways, including increasing membrane permeability in leaves, damaging sub-cellular membranes such as thylakoid membranes, thus impeding light harvesting, electron transport rates and ATP generation [29–32]. Under HS the primary carbon-fixing enzyme, rubisco, is also more active as an oxygenase leading to the production of 2-phosphoglycolate, which is eliminated through the photorespiratory pathway resulting in partial loss of previously fixed carbon [4, 33]. Altogether, the reduced photosynthetic efficiency and increased respiration-photorespiration rates due to HS alter the dynamics between source and sink organs, leading to yield decline [34].

The capacity of primary source tissue to mobilize photoassimilates and the ability of sink tissue (grain) to accumulate the transported sugars determines the extent of grain filling [18, 19]. A significant proportion of the assimilates accumulating in the grains are derived from the upper canopy [35–37]. One estimate suggests that the youngest three leaves may contribute over 50% of the assimilates into the rice grain filling pool [38]. While foliar tissue is the primary source of photoassimilates, non-foliar tissue such as developing rice panicles that stay green during the grain filling period are also photosynthetically active and contributes toward photoassimilate accumulation in grains [39, 40]. Previous studies have stated that the contribution of green inflorescence tissues to carbon assimilation (*A*_*gross*_) is equivalent to ~ 30% of the flag leaf [41–43]. Additionally, it has been reported that non-foliar organs exhibit different photosynthetic characteristics than foliar organs [41]. Currently, determining the dynamic relationship between foliar and non-foliar organs remains elusive. Given the importance of non-foliar organs in contributing to the grain assimilate pool, the effect of HS on their net photosynthetic contributions remains unexplored.

The temporal evaluation of foliar photosynthetic parameters on a per unit area basis can be accomplished non-destructively using well-established protocols. Instrumentation for these experiments is designed for laminar leaf surfaces for which precise surface areas can be determined. However, the measurement of non-laminar organs (inflorescence/panicle) with their intricate and complex architectures is challenging [44]. Resolving this issue was recently attempted [20], where destructive and 2D approaches were followed for calculating panicle area. However, 2D projections for the 3D rice panicle (globular structure) can result in a substantial loss of spatial information [45]. It is an imprecise estimation of the projected area for a 3D structure and does not accurately assess the panicle-based gas exchange parameters on a per unit area basis. Further, the destructive sampling of the panicles is subjective, laborious, and does not allow the estimation of photosynthetic dynamics of developing Query ID="Q9" Text="References: As per pubmed findings, citation details [Page no and volume id] for Reference [19] have been inserted. Kindly check and confirm the inserted details." panicles in a temporal manner. Recent advances in image-based plant phenotyping have enabled the development of a 3D-panicle imaging platform (*PI-Plat*) for high-resolution, temporal assessment of vegetative and inflorescence-related traits in a non-destructive and precise manner [46–48]. Digital traits derived from 3D reconstructed panicles are more sensitive and accurate than results from 2D images [46]. Thus, the non-destructive estimation of panicle size parameters in rice using 3D-imaging platforms can be used to establish surface area normalized panicle photosynthetic assessments.

We combined panicle surface area measurements with a customized gas Query ID="Q11" Text="References: As per pubmed findings, citation details [Publisher location] for Reference [29] have been inserted. Kindly check and confirm the inserted details." exchange cylinder that allowed unrestricted enclosure of panicles, thus overcoming a major limitation of shading as reported in other studies [20, 39, 49]. Measuring flag leaf and panicle photosynthetic parameters concurrently enabled us to identify relationships between foliar and non-foliar tissue gas exchange rates under control and HS conditions. This novel approach was able to identify changes in source-sink dynamics in response to HS, as well as the differential response of two temperate japonica rice accessions that were previously known to differ in their sensitivity to HS during grain development (GSOR Ids: 301110, TEJ-1 and 301195, TEJ-2) [12]. Our results establish a viable method for a more precise temporal evaluation of source-sink relationships during reproductive development, in response to HS, for the study of genetic diversity in photosynthetic strategies among rice accessions. Although we specifically examined HS response, the method should also be useful under other stress conditions as well.

## Results

### Heat stress induces differential morphological responses in panicles

**Fig. 1:**
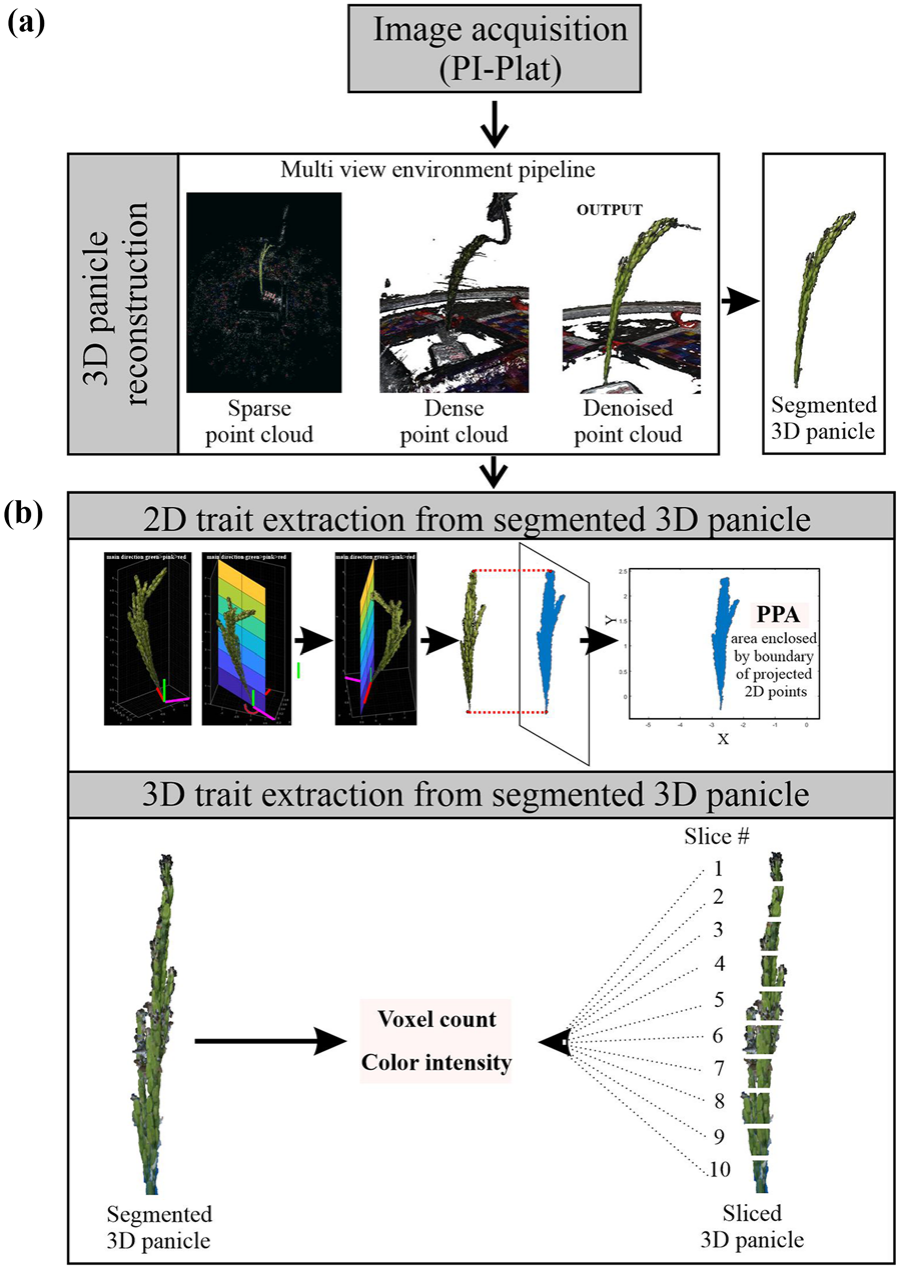
3D imaging acquisition and analysis workflow. **a** Workflow for the reconstruction of 3D panicle from Multiview images using PI-Plat imaging platform. **b** Trait extraction from the reconstructed 3D panicle. The upper panel shows the extracted projected panicle area (PPA) from the boundary of projected 2D points. The lower panel shows the traits derived from the segmented 3D panicle and sliced 3D panicle (voxel count and color intensity). Slice 1 corresponds to the top-most slice and slice 10 corresponds to the bottom-most slice of the 3D panicle

**Fig. 2:**
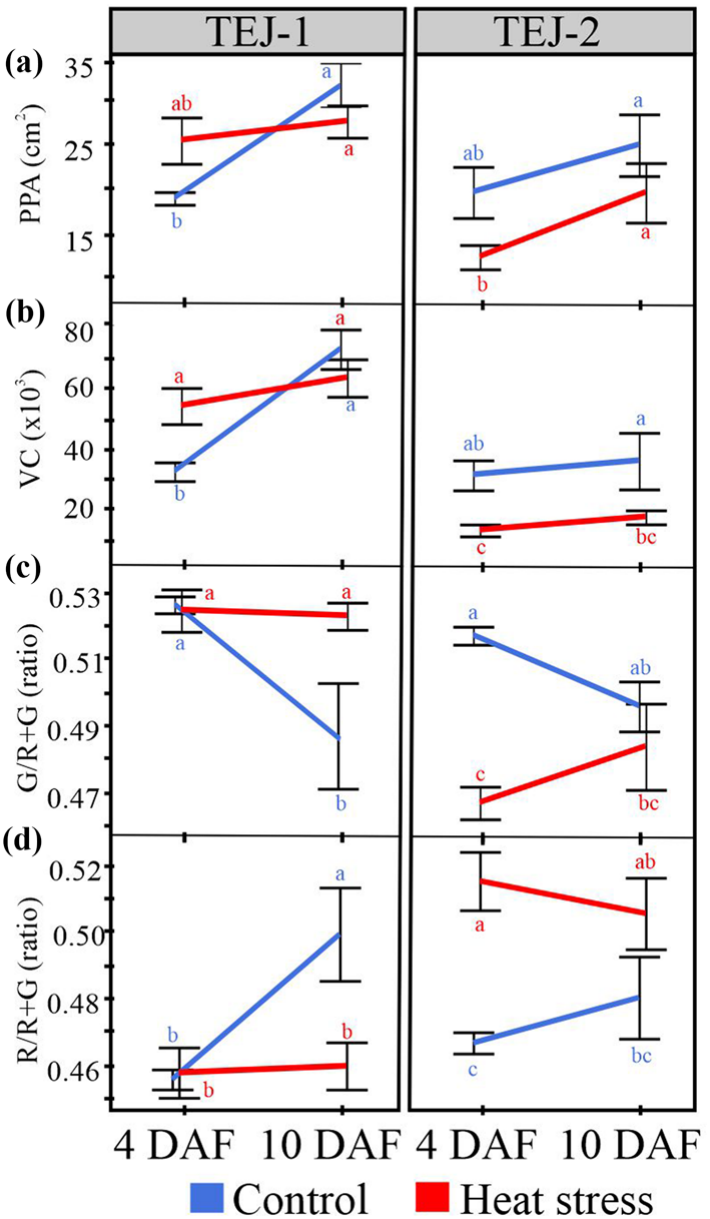
Digital trait analysis from 3D reconstructed panicles of TEJ-1 and TEJ-2. **a** PPA (Projected panicle area) in cm^2^, **b** VC (Voxel count) representing the point count in a 3D plane, **c** Ratio of green pixels (G) to the sum of red and green pixels (R + G) in a 3D plane, **d** Ratio of red pixels (R) to the sum of red and green pixels (R + G) in a 3D plane; are plotted. Left and right panel represents TEJ-1 and TEJ-2, respectively. *n* = 3–4 plants per data point. For statistics, student’s t-test was conducted separately for each genotype to compare each temperature treatment between the time points. Significant differences are indicated by different letters. Error bars represent ± SE

The purpose of this study was to establish whether multi-view images captured by using PI-Plat could be combined with a novel method for whole panicle gas exchange measurements to follow photosynthetic dynamics during reproductive development. We imposed a moderate HS for 4 or 10 days beginning 1 day after fertilization (DAF) and measured the photosynthetic response of both foliar and panicle tissue under control (28/23 °C; day/night) and HS (36/32 °C; day/night). Two rice lines (TEJ-1 and TEJ-2) genetically diverse in their response to HS were compared. From captured images from multiple angles 3D point clouds of panicles were reconstructed to extract the digital traits of a panicle (Fig. 1). The derived digital traits included, projected panicle area (PPA), voxel count (VC), and color intensity (red and green pixels) representing the panicle’s area, volume, and green/red pixel proportion, respectively [24, 25, 46]. We first used the digital traits to examine whether they could distinguish temporal differences in inflorescence architecture due to HS, and then whether the response differed between TEJ-1 and TEJ-2 (Fig. 2). In TEJ-1, PPA exhibited an increase from 4 to 10 DAF under control conditions, while no significant change was observed under HS (Fig. 2a). An increase in PPA was also observed in TEJ-2 from 4 to 10 DAF under control conditions. However, unlike TEJ-1, TEJ-2 exhibited an increase in PPA from 4 to 10 DAF under HS (Fig. 2a). VC also exhibited a similar trend as PPA in both the genotypes under control and HS (Fig. 2b). For downstream panicle level gas exchange, we decided to use PPA as the normalizing parameter. The PPA and VC for TEJ-2 were lower than for TEJ-1 under both temperature conditions, confirming our direct observation of TEJ-2 having a smaller panicle than TEJ-1 (Fig. 2a and b).

### Panicle photosynthetic response to heat stress is dynamic

**Fig. 3:**
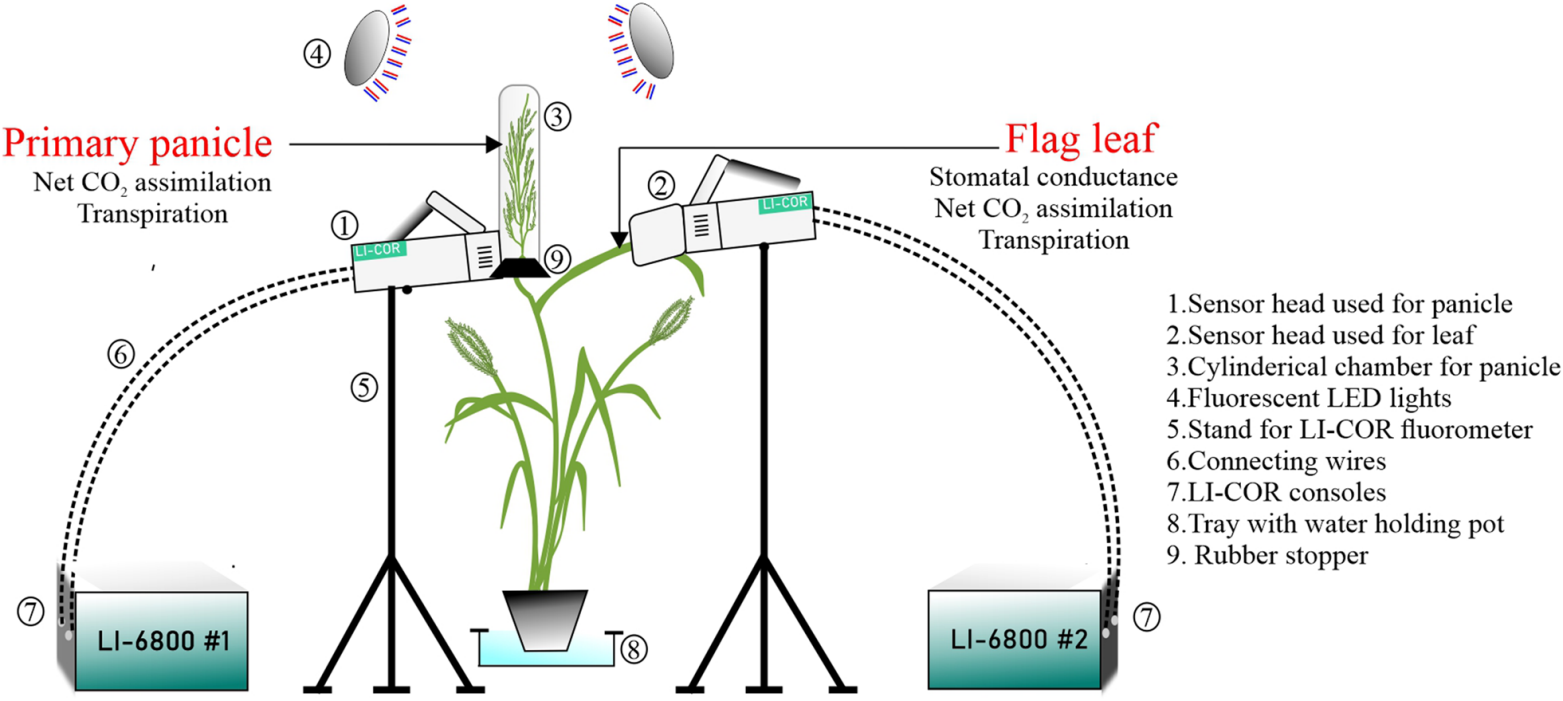
Pictorial representation of the setup used for measuring gas exchange parameters Gas exchange parameters for flag leaf and primary panicle were simultaneously measured using two LI-6800 devices. The photosynthetic parameters obtained from leaf and panicle that are used for comparative analysis in the study are mentioned in the picture. Numbers represent details of each part of the setup

**Fig. 4:**
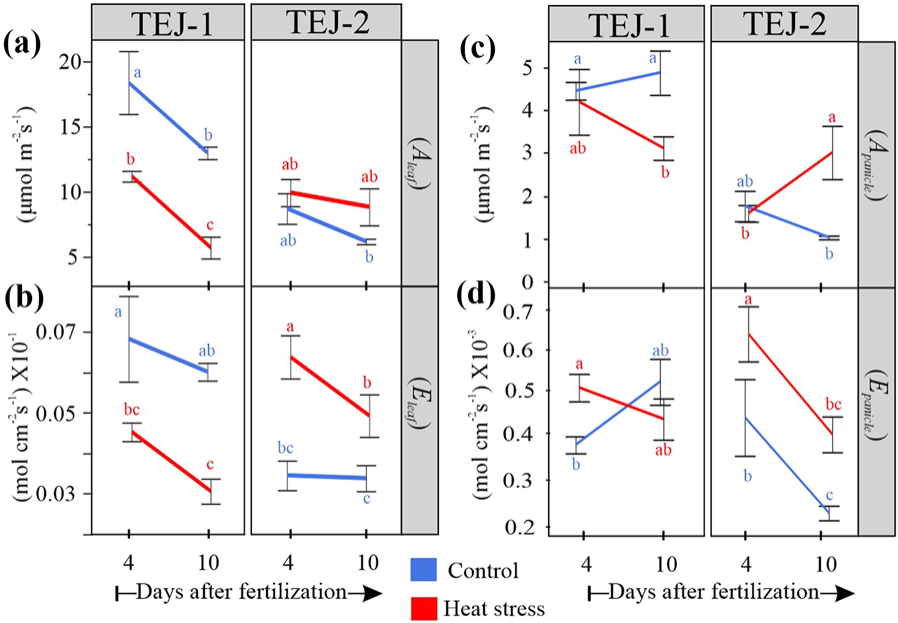
Gas exchange parameters for TEJ-1 and TEJ-2. **a** Flag leaf assimilation, **b** Flag leaf transpiration, **c** panicle assimilation, and **d** panicle transpiration parameters for TEJ-1 and TEJ-2 under control and heat stress conditions at 4 and 10 DAF (A: net CO_2_ assimilation; E: transpiration). *n* = 3–4 plants per data point. For statistics, student’s t-test was conducted separately for each genotype to compare each temperature treatment between the time points. Significant differences are indicated by different letters. Error bars represent ± SE

We next determined whether the gas exchange response of the primary panicle and its corresponding flag leaf varied under the conditions described above (Fig. 3). A standard leaf chamber of the open infra-red gas analyzer was used for the flag leaf. The foliar and non-foliar photosynthetic measurements were conducted the same day as the panicle imaging. For TEJ-1 we observed significantly lower (*p* < 0.001) stomatal conductance (*gsw*_*leaf*_) for the flag leaf under HS compared to controls at both the time points (4 and 10 DAF) (Additional file 4). In contrast, flag leaf of TEJ-2 exhibited higher *gsw*_*leaf*_ at both 4 and 10 DAF under HS (Additional file 4). Since apparent transpiration rate (*E*) is a function of stomatal conductance, *E*_*leaf*_ also remained significantly lower (p < 0.001) for the TEJ-1 plants grown under HS at both time points compared to controls (Fig. 4a). TEJ-2 plants had higher *E*_*leaf*_ under HS (Fig. 4a). Consistent with stomatal conductance (*gsw*_*leaf*_*)*, recorded carbon assimilation (*A*_*leaf*_) was significantly lower (p < 0.001) for TEJ-1 plants under HS at both the time points (Fig. 4a). The carbon assimilation (*A*_*leaf*_) rate of TEJ-2 did not change significantly under HS at 4 and 10 DAF (Fig. 4a). Furthermore, we observed that leaf water use efficiency (*WUE*_*leaf*_) in TEJ-1 was significantly less under HS than control at both 4 and 10 DAF, with a decreasing trend (Additional file 9). In contrast, TEJ-2 exhibited an increasing trend for *WUE*_*leaf*_ in HS and a decreasing trend in control from 4 to 10 DAF (Additional file 9). This data suggest that TEJ-1 exhibits greater gas exchange sensitivity in foliar tissue to HS relative to TEJ-2.

We next measured the panicle level photosynthetic response of TEJ-1 and TEJ-2 under HS using a custom-built LI-6800-compatible cylindrical chamber for panicle measurements (Fig. 3). We used PPA for normalizing panicle measurements across genotypes and treatments on a per unit area basis (Fig. 1b). In TEJ-1, there was no difference between *A*_*Panicle*_ under control and HS at 4 DAF (Fig. 4b). However, the *A*_*Panicle*_ was reduced under HS at 10 DAF in TEJ-1. Like TEJ-1, no difference in *A*_*Panicle*_ under control and HS was observed at 4 DAF in TEJ-2 (Fig. 4b). Notably, in TEJ-2, the *A*_*Panicle*_ was higher under HS than control at 10 DAF (Fig. 4b-upper part). The panicle level apparent transpiration rates (*E*_*Panicle*_) were higher under HS than control at 4 DAF in both accessions (Fig. 4b). At 10 DAF, the apparent transpiration rate was similar under HS and control in TEJ-1, and higher under HS than control in TEJ-2 (Fig. 4b-lower part). Additionally, panicle water use efficiency (*WUE*_*panicle*_*)* of TEJ-1 under HS remained significantly lower than control at both the timepoints (Additional file 9). However, *WUE*_*panicle*_ of TEJ-2 exhibited a significant increase at 10 DAF under HS than control (Additional file 9). These photosynthetic measurements indicate that TEJ-1 and TEJ-2 have contrasting responses under HS for *A*_*leaf*_ and *A*_*panicle*_ at 10 DAF. Further, the percent change observed in *A*_*leaf*_ and *A*_*panicle*_ under HS when compared to corresponding controls at 10 DAF (Additional file 5) quantified this genotypic difference. At 10 DAF, *A*_*leaf*_ and *A*_*panicle*_ were reduced by 56% and 26%, respectively, in TEJ-1 under HS compared to their corresponding controls. In contrast, in TEJ-2, *A*_*leaf*_ and *A*_*panicle*_ increased by 57% and 121% respectively, under HS relative to controls (Additional file 5). Collectively, these analyses indicate the potential of our experimental approach involving concurrent measurement of foliar and non-foliar photosynthetic parameters to discern genotypic differences for photosynthetic parameters under heat stress.

Further, we investigated if the panicle-level photosynthetic parameters measured using the cylinder-based chamber can be estimated from the digital traits extracted from the 3D reconstructed panicles. For this, we extracted the pixel color intensities from 3D-reconstructed panicles to differentiate their response to HS. The 4 and 10 DAF measurements correspond to the active grain filling phase when the panicle is predominantly green. Since green (G) pixel intensity can be used as a proxy for panicle surface chlorophyll content, we estimated the proportion of green pixels to the sum of red and green pixels [G/(R + G)] to determine changes in response to HS. Under control conditions, TEJ-1 exhibited a decline in green pixel proportion from 4 to 10 DAF (Fig. 2c). While under HS, no significant decline was observed from 4 to 10 DAF in green pixel ratio in TEJ-1 (Fig. 2c). The proportion of green pixels decreased from 4 to 10 DAF in TEJ-2 under control conditions (Fig. 2c). These observations did not explain the change or lack of change in photosynthetic parameters for both genotypes under control conditions. However, the proportion of green pixels increased from 4 to 10 DAF in TEJ-2 under HS (Fig. 2c). This observation was consistent with the striking increase observed in *A*_*panicle*_ in TEJ-2 at 10 DAF under HS (Fig. 4b-upper part). As the panicle approaches maturity, pixels are expected to shift towards R. Therefore, we also analyzed the proportion of red pixels to the sum of red and green pixels [R/(R + G)]. In TEJ-1, the proportion of red pixels increased from 4 to 10 DAF under control conditions, while it remained similar between 4 and 10 DAF under HS (Fig. 2d). TEJ-2 also exhibited a similar trend as TEJ-1 for red pixels proportion under control conditions (Fig. 2d). However, the red pixel proportion was higher in TEJ-2 than TEJ-1 under HS at both time points (Fig. 2d). Based on our analysis, whole panicle level G pixel proportion does not correspond well with panicle gas exchange measurements.

### Digital slicing of reconstructed panicles captures panicle level spatial variation

**Fig. 5:**
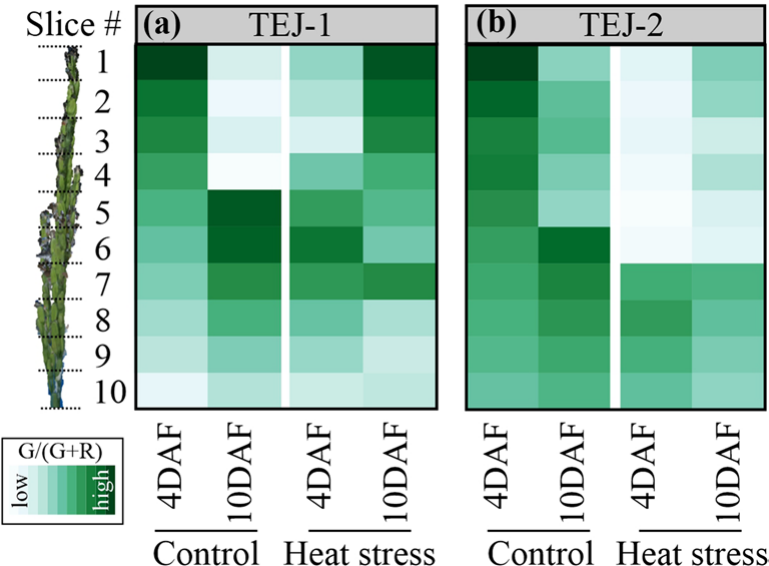
Spatial pattern of green pixel intensity for TEJ-1 and TEJ-2. Shifts in green pixel intensity resolved into 3D slices using the panicle point cloud **a** TEJ-1 and **b** TEJ-2. Progression of color from white to green in the heat map represents an increase in green pixel intensity, which is a proxy for the chlorophyll content of the panicle surface. N = 3–4 plants per data point. Respective values from each slice of all the replicates were averaged to make the final heat map. Control and heat stress values of green pixel intensity for the genotypes are on the same scale to show the temporal and spatial changes

The observed inconsistency between photosynthetic parameters and green pixel proportion promoted us to further examine the pixel color intensities by accounting for spatial variability along the panicle length due to the variable developmental stage of the seeds, resulting from asynchronous fertilization. Therefore, we divided the 3D reconstructed panicle into ten equal slices. Digital traits were obtained for individual slices (Fig. 1) and compared between control and HS for each genotype (Fig. 5). We performed spatial analysis for VC and green pixel proportion [G/(R + G)] for both genotypes (Fig. 5 and Additional file 7). In TEJ-1, a gradient in green pixel proportion was observed from top slices (slices 1–4) having higher green pixel proportion than lower slices (slices 5–10) at 4 DAF under control conditions (Fig. 5a). By the 10 DAF time point, the top slices (slices 1–4) had reduced green pixel proportion and the bottom slices (slices 5–10) had increased green pixel proportion under control conditions in TEJ-1 (Fig. 5a). Unlike control conditions, a gradient in green pixel proportion was observed with middle slices (slice 4–7) having higher proportion, followed by bottom slices (slices 8–10), and then the top slices (slices 1–3) at 4 DAF under HS in TEJ-1 (Fig. 5a). The green pixel proportion of upper slices (slices 1–4) increased at 10 DAF compared with 4 DAF, whereas they were lower for most of the bottom slices (slices 5–10; except slice 7) under HS in TEJ-1 (Fig. 5a). TEJ-2 also had a gradient in green pixel proportion under control conditions at 4 DAF with the top slices (slices 1–4) having higher green pixel proportion than the bottom slices (slices 5–10) (Fig. 5b). At 10 DAF, the top slices (slices 1–4) had a reduced green pixel proportion, while the bottom slices (slices 5–10) had similar green pixel proportions as those of 4 DAF under control conditions in TEJ-2 (Fig. 5b). A notable feature of the TEJ-2 under HS was its ability to largely maintain a higher green pixel proportion for the bottom slices (slices 7–10) at 4 and 10 DAF relative to control values (Fig. 5b). At 10 DAF in TEJ-2, the green pixel proportion for top slices (slices 1–6) increased slightly compared to 4 DAF under HS (Fig. 5b). Collectively, variations in the green pixel proportion pattern obtained from slicing of 3D panicles illustrates the spatial heterogeneity among the florets and its transition with progression of both development and stress duration.

### Correlations between digital traits and photosynthetic measurements vary with genotypes

**Fig. 6:**
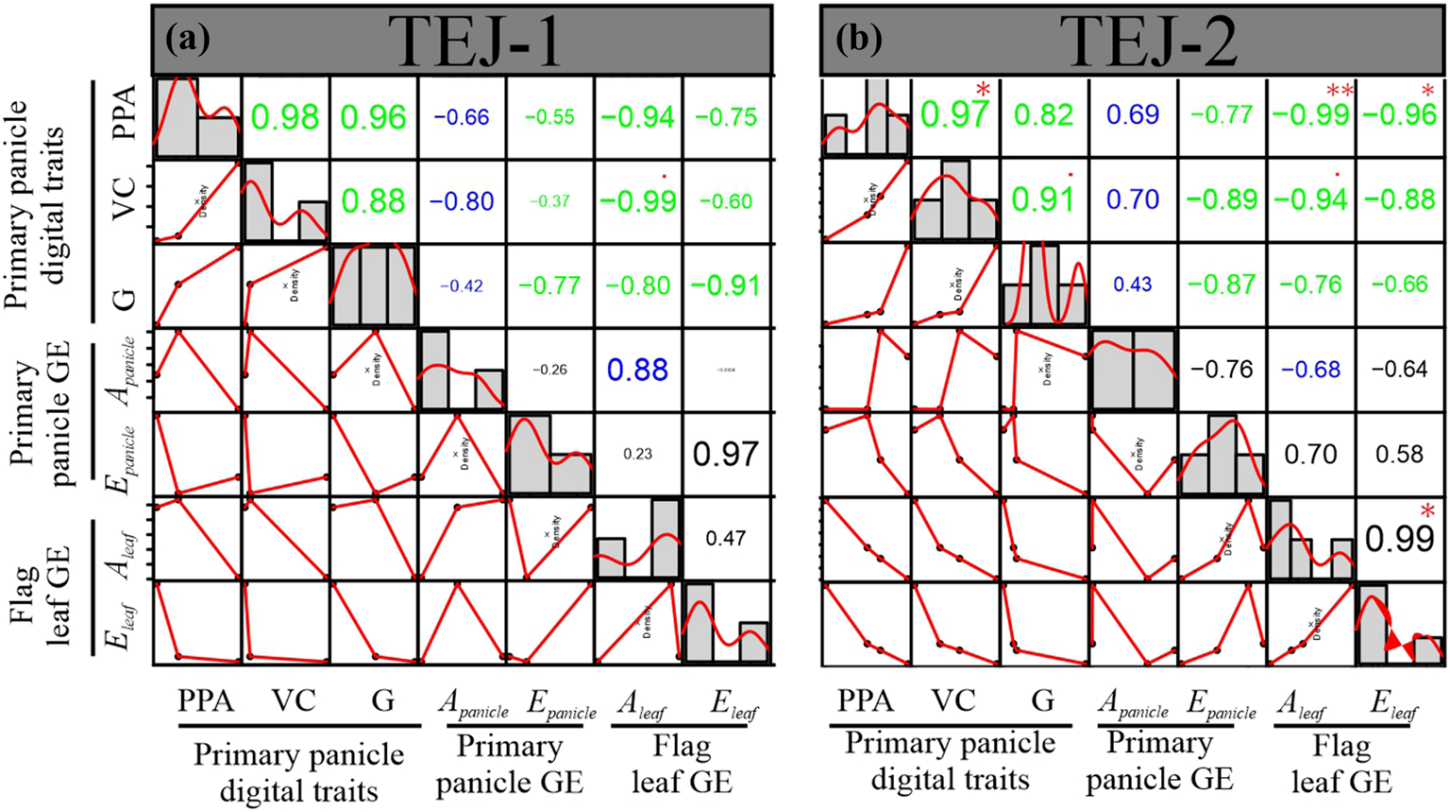
Correlation analysis. Correlation of primary panicle digital traits, primary panicle gas exchange (GE) parameters and flag leaf GE parameters at 10 DAF under HS in **a** TEJ-1 and **b** TEJ-2. Histograms and red lines represent each trait’s distribution. Green-colored text indicates a similar type of correlation in TEJ-1 and TEJ-2 for the respective traits. Blue-colored text represents contrasting correlation values in TEJ-1 and TEJ-2 for underlying traits. The font size of the text for correlation value is proportional to the actual value of the correlation between the parameters being compared. *PPA* projected panicle area, *VC* voxel count, *G* proportion of green pixel intensity, *A*_*panicle*_ Net CO_2_ assimilation of primary panicle, *E*_*panicle*_: Transpiration of primary panicle, *A*_*leaf*_: Net CO_2_ assimilation of flag leaf, *E*_*leaf*_: Transpiration of flag leaf, *GE* gas exchange. (**p < 0.01; *p ≤ 0.05.)

We next examined the relationship among 3D reconstruction-derived features and photosynthetic parameters for the genotypic responses to HS at 10 DAF. We selected the 10 DAF for this analysis as we observed the most significant genotypic contrast at this time point under HS. We used the digital traits (PPA, VC, and G) and photosynthetic measurements *(*A_*panicle*_*,* E_*panicle*_*,* A_*leaf*_*,* E_*leaf*_*)* to perform pairwise correlation analysis separately for TEJ-1 and TEJ-2 under HS. The derived digital traits, i.e., PPA, VC, and green pixel proportion, showed a strong positive correlation among themselves and a negative correlation with A_*leaf*_, A_*panicle*_, and E_*leaf*_ in both genotypes (Fig. 6, green boxes). Further, the correlation between some of the examined parameters exhibited contrasting values in TEJ-1 and TEJ-2 (Fig. 6, blue boxes). Although these correlation values between particular digital traits and photosynthetic parameters were not statistically significant, they still suggest a divergent response for TEJ-1 and TEJ-2 under HS. For instance, in TEJ-1, the correlation of *A*_*panicle*_ with PPA, VC, and G was −0.42, −0.80, and −0.66, respectively (Fig. 6a, blue boxes), while in TEJ-2, the correlation of *A*_*panicle*_ with PPA, VC, and G were + 0.43, + 0.70, and + 0.69, respectively (Fig. 6b, blue boxes). These results suggest that despite having a larger panicle size and higher pixel count under HS, TEJ-1 does not exhibit an increase in *A*_*panicle*_, resulting in negative correlation values. In TEJ-2, *A*_*panicle*_ increases along with PPA, VC, and G under HS, resulting in a positive correlation. Further, the correlation between *A*_*panicle*_ and *A*_*leaf*_ in TEJ-1 and TEJ-2 was + 0.88 and −0.68, respectively (Fig. 6, blue box). These results suggest that in TEJ-1, both *A*_*panicle*_ and *A*_*leaf*_ are decreasing under HS, leading to a positive correlation value (Figs. 2 and 6), while TEJ-2 has higher *A*_*panicle*_ and more stable *A*_*leaf*_ under HS, resulting in negative correlation (Figs. 2 and 6).

### Analysis of mature grain parameters of TEJ-1 and TEJ-2 under HS

**Fig. 7:**
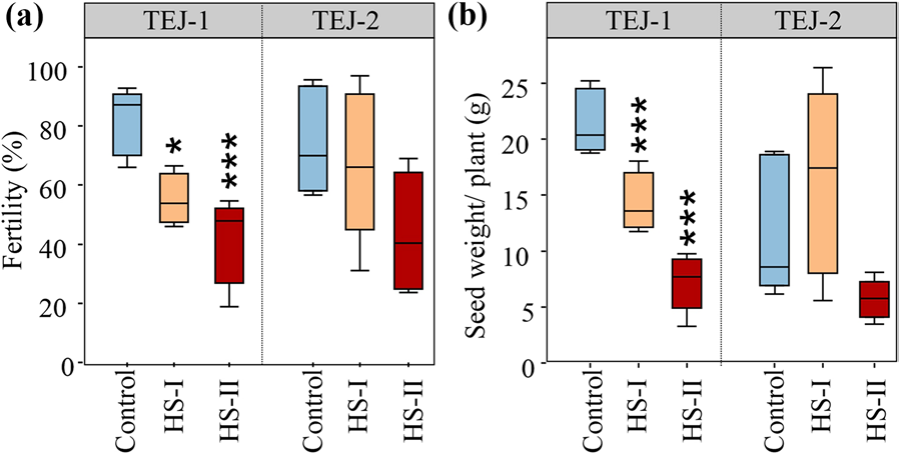
Mature grain physiological parameters for TEJ-1 and TEJ-2. Impact of heat stress on mature seeds at whole plant level in TEJ-1 and TEJ-2 developing under control and heat stress (HS) conditions during grain filling. HS-I and HS-II refer to the duration of imposed HS i.e., 1–4 DAF (HS-I) and 1–10 DAF (HS-II). **a** Quantification of spikelet fertility (%) and **b** seed weight in grams at the whole plant level evaluated at the time of physiological maturity. Box plots show the median and the upper quartiles and black dots signify outliers (5th/95th percentile). N = 1500–3500 seeds from 4–6 plants per data point. For statistics, t-test was used to compare heat stressed mature seeds with respective controls (***p < 0.001; *p ≤ 0.05.)

The digital traits from 3D reconstructed panicle and photosynthetic measurements indicate that TEJ-1 and TEJ-2 have a differential response to HS. We next asked if these observed differences at early seed development stages translate to differences in grain traits at maturity. For this, we imposed short (HS-I; 2–4 DAF) and long (HS-II; 2–10 DAF) duration HS and measured seed length, width, weight, and fertility (Additional file 6a). Mature grain parameters of TEJ-1 and TEJ-2 did not differ significantly different between control and HS-I, except for fertility (%), which was higher in TEJ-2 after heat treatment (Additional file 6b). Under HS-II, fertility was significantly reduced in TEJ-1 but not in TEJ-2 (Additional file 6b). Seed length was not affected in TEJ-1 but increased under HS-II in TEJ-2. A significant reduction in seed weight and width of marked seeds on the primary panicles was observed for both TEJ-1 and 2 at HS-II compared to respective controls (Additional file 6b). The results indicate that TEJ-1 and 2 exhibited differential tolerance to the longer duration heat stress (HS-II) for marked seeds. At the whole plant level, the fertility and per plant grain weight were reduced due to HS-I and HS-II in TEJ-1 compared to its control (Fig. 7a). However, these two parameters were not affected for both heat treatments in TEJ-2. Additionally, we observed no significant difference in panicle length for the two genotypes in either of the temperature conditions (Table 1). The number of panicles was similar for each genotype in all the temperature conditions. However, TEJ-1 exhibited a significantly higher number of panicles than TEJ-2 in general. Despite having a greater number of panicles, the number of filled spikelets per panicle was significantly reduced in TEJ-1 under HS-II, possibly due to reduced grain filling. Interestingly, in TEJ-2, the filled spikelet number per panicle was significantly higher than TEJ-1 and no significant impact of HS was observed (Table 1). The whole plant level seed trait data suggests that TEJ-2 exhibits greater heat tolerance even for seeds that were fertilized under heat stress compared to TEJ-1. The marked seeds are distinct from whole plant level seeds as they are derived from fertilization events that occur before the imposition of HS treatments.

**Table 1:** Table representing panicle length (cm), number of panicles, and number of filled spikelets per panicle in TEJ-1 and TEJ-2 under control, HS-I, and HS-II conditions

Genotype	Treatment	Panicle length (cm)	Number of panicles	Number of filled spikelets per panicle
TEJ-1	Control	17.5 ± 0.56^a^	14.8 ± 1.14^a^	56.6 ± 7.7^a^
TEJ-1	HS-I	17.6 ± 0.59^a^	12.2 ± 1.28^a^	46.3 ± 8.6^ab^
TEJ-1	HS-II	17.3 ± 0.57^a^	12.2 ± 1.14^a^	29.8 ± 6.7^c^
TEJ-2	Control	16.0 ± 0.50^a^	5.5 ± 1.5^b^	62.8 ± 9.9^a^
TEJ-2	HS-I	16.1 ± 0.56^a^	6.6 ± 1.04^b^	62.8 ± 7.04^a^
TEJ-2	HS-II	16.3 ± 0.57^a^	6.1 ± 1.47^b^	48.4 ± 9.9^ab^

Student's t-test was conducted to compare the genotypes and treatments for each of these parameters separately. Different letters indicate significant differences among the comparisons for each parameter (parameters connected by the same letter are not statistically significant). N = 4–6 plants per data point and ± represents standard error

## Discussion

It is likely that the negative effects of HS on seed development results partially from a disturbance in photosynthesis not only in foliar tissues, but also in non-foliar tissues, as well as from the dynamic interactions between these two photosynthate sources. To explore these questions, we developed and tested a novel and more precise method to non-destructively measure rice panicle photosynthetic parameters. We hypothesized that this approach, combined with concurrent foliar measurements by traditional methods, would enhance our understanding of the photosynthetic response to HS. Further, we postulated that this method could uncover differences between rice lines that differ in their HS response during reproductive development. Such comparative analyses could eventually help explain why grain fill in some rice accessions is less affected by HS than others. We determined the relative rates of gas exchange between flag leaf and panicle under HS during the grain filling stage, the effect of altered carbon fixation (of flag leaf and panicle) due to HS on the final grain yield parameters and distinguish the differential physiological response of two genotypes under HS. In addition to photosynthetic measurements, we also assessed panicle level digital traits to track developmental dynamics along the panicle length under control and HS conditions. For this, we digitally partitioned the 3D reconstructed panicle into ten equal slices and extracted digital traits for each slice. The spatial perspective of the 3D reconstructed panicle enabled us to discern differences between TEJ-1 and TEJ-2 heat stress response at greater resolution (Fig. 5). The analysis of voxel count (VC) and projected panicle area (PPA) from the whole panicles indicated an increasing trend from 4 to 10 DAF in both genotypes under optimal conditions (Fig. 2a and b). The spatiotemporal characterization of the panicle slices was able to differentiate responses of the two genotypes that were not evident from whole panicle traits. For instance, the whole 3D panicle of TEJ-1 under HS did not exhibit a significant change in the green pixel proportion from 4 to 10 DAF (Fig. 2c). However, sliced 3D panicle results indicate that the green pixel proportion at 4 DAF was higher for proximal slices whereas at 10 DAF higher for distal slices (Fig. 5a). This distinctive spatial distribution of green pixels at 4 and 10 DAF explains why the overall green pixel proportions were not changed under HS for whole 3D panicle of TEJ-1. For TEJ-2, we observed more stable green pixel spatial profile when comparing the 4 and 10 DAF under HS. TEJ-2 slicing results show that the proximal panicle slices (slices 7–10) do not exhibit a drop in the green pixel intensity at 10 DAF under HS (Fig. 5b). This is in contrast with the proximal slices (8–10) in the TEJ-1 at 10 DAF. It is plausible that the observed increase in *A*_*panicle*_ at 10 DAF under HS in TEJ-2 could be primarily due to proximal spikelets that “stay green” for a longer duration. Alternatively, the panicle architecture of TEJ-2 may be different from TEJ-1 in maintaining growth in proximal part, reflected in largely stable values across time and treatments.

The digital traits derived from 3D reconstructed panicles were able to detect variations in the developmental progression of the two genotypes under HS. Since developing grain acts as the active sink tissue, the progression in grain development depends upon the accumulation and utilization of the photoassimilates. To examine the source-sink relationship and its effect on grain development, we measured photosynthetic parameters for the flag leaf and primary panicle simultaneously. Apart from the major photosynthetic parameters impacting carbon fixation, parameters like vapor pressure deficit (*VPD*) are known to increase under HS, and hence are a factor for consideration [50, 51]. Our results show a higher leaf *VPD* for the plants exposed to HS, indicating a greater leaf-atmosphere diffusion gradient (Additional file 4: Figure S4). At higher *VPD*, plants tend to lose more water and trigger stomatal closure to maintain plant water status under limited water conditions [4, 50, 51]. However, if water availability and *VPD* are not restrictive factors, high temperature can induce guard cell expansion which facilitates stomatal opening to trigger evaporative cooling of the leaf [^52^, ^53^, ^54^]. The two genotypes in this study showed a contrasting response in foliar gas exchange parameters on exposure to HS under similar growth conditions, including water availability and *VPD* (Fig. 4 and Additional file 4). For instance, a reduction in leaf stomatal conductance, apparent transpiration rate, and carbon assimilation was observed in TEJ-1 under HS even though plants were growing in well-watered conditions. In contrast, TEJ-2 maintains a higher apparent transpiration rate, stomatal conductance, and carbon assimilation under longer duration HS, suggesting that there may be a temperature-dependent or independent stomatal response difference between the two genotypes. This could be due to genotypic variation in biomechanical elasticity of the guard cell complex. Alternatively, TEJ-1 may lack the hydraulic structure to sustain water movement under high *VPD* conditions, resulting in differential ABA accumulation in the guard cells.

One of the limitations of using LI-6800 equipped with customized cylinders for assessing gas exchange parameters is that it cannot measure stomatal conductance (*gsw*_*panicle*_). However, the other non-foliar, panicle-based photosynthetic measurements indicated that net CO_2_ assimilation (*A*_*panicle*_) for both genotypes was similar between optimal and HS conditions at 4 DAF (Fig. 4b). However, *A*_*panicle*_ exhibited a contrasting response in TEJ-1 and TEJ-2 under HS at 10 DAF. TEJ-1 showed a decline and TEJ-2 showed an increase in *A*_*panicle*_ under HS compared to their respective controls at 10 DAF (Fig. 4b). Notably, the apparent transpiration rate for the TEJ-2 declined under HS but the *A*_*panicle*_ increased for 10 DAF panicles. Therefore, the estimated WUE for TEJ-2 was also significantly higher than the optimal conditions at 10 DAF (Additional file 9). This decoupling of *A*_*panicle*_ from the apparent transpiration rate in TEJ-2 under HS is intriguing as it likely promotes carbon assimilation rather than evaporative cooling of the panicle.

The photosynthetic parameters measured for two genotypes were consistent with plant-level grain parameters. For instance, TEJ-1, which exhibited a decline in assimilation rate (*A*_*panicle*_ and *A*_*leaf*_) measured during the grain filling stage, also had significantly reduced mature grain weight and fertility parameters (Figs. 4 and 7). TEJ-2 had an enhanced assimilation rate (*A*_*panicle*_ and *A*_*leaf*_) under HS at 10 DAF and showed no significant change in mature grain weight and fertility parameters at the whole-plant level (Figs. 4 and 7). In TEJ-1, there was a greater percent decrease in *A*_*leaf*_ (-57%) than in *A*_*panicle*_ (-26%) at 10 DAF under HS as compared to respective controls. In contrast, in TEJ-2 the percent increase in *A*_*leaf*_ (57%) was considerably less than in *A*_*panicle*_ (121%) in response to HS relative to control values. The higher *A*_*panicle*_ for TEJ-2 under HS at 10 DAF is also consistent with the more stable spatial profile of TEJ-2 for green pixel proportion under HS relative to TEJ-1, especially in the proximal end of panicles. Furthermore, the impacted mature seed weight and fertility parameters for TEJ-1 under HS explains that despite of acquiring green pixels during the active grain filling phase, reduced *A*_*panicle*_ resulted into the compromised grain filling capacity (Figs. 4, 5, and 7).

Overall, this study provides a non-destructive methodology to determine foliar and non-foliar gas exchange parameters in rice. The presented method demonstrates its capability of distinguishing two genotypes based on the photosynthetic capacity of their source-sink organs during the grain development period. Further, this method will aid future studies aiming at characterizing the genotypic differences between the source-sink relationships and non-foliar photosynthesis on a large scale as well as in a variety of crops bearing compact inflorescences.

## Conclusion

This work shows the potential value of combining foliar and non-foliar physiological measurements to examine dynamic heat stress response in rice, and to identify genotypic differences in this response. By measuring temporal dynamics along the panicle length, we were also able to discern spatial differences under heat stress. This improved non-destructive approach combines 3D imaging, photosynthetic measurements, and grain physiology, and could be used to gain a spatiotemporal perspective on multiple stress responses and in a variety of cereal species bearing compact inflorescences.

## Materials and methods

### Plant material and growth conditions

Two temperate japonica rice genotypes, GSOR Ids: 301110 (TEJ-1) and 301195 (TEJ-2), were selected based on their heat stress (HS) response as observed in our previous study, [12]. Mature seeds from the two accessions were dehusked using a Kett TR-130, sterilized with water and bleach (40%, v/v), and rinsed with sterile water. The seeds were germinated in the dark on half-strength Murashige and Skoog media. After 5 days, germinated seedlings were transplanted and grown under controlled greenhouse conditions: 16 h light and 8 h dark at 28 ± 1 °C and 23 ± 1 °C, respectively. Relative humidity ranged from 55 to 60% throughout the experiments.

### Temperature treatments

A set of 10 plants per genotype were used to do the PI-Plat imaging and record photosynthetic measurements using the LI-6800 (Li-Cor Inc., Lincoln, NE). The methodological details about imaging and photosynthetic measurements are discussed in the sections below. All plants were grown under controlled conditions until flowering. For each genotype, upon ~ 50% completion of primary panicle flowering, half of the plants were kept under control conditions (16 h light and 8 h dark at 28 ± 1 °C and 23 ±  °C), while the other half were moved to a greenhouse set-up for moderate heat stress (HS) treatment (16 h light and 8 h dark at 36 ± 1 °C and 32 ± 1 °C) (Additional file 1a). A 36 °C day and 32 °C night heat stress treatment is results in reduced seed size and impacts seed development as the critical temperature threshold for rice is 35 °C during reproductive development. Primary panicle imaging and photosynthetic measurements were recorded from the plants growing separately under control and HS conditions at two time points i.e., 4 and 10 d (Additional file 1a). For experimental accuracy, the primary panicle was used for photosynthetic measurements along with the flag leaf.

Another set of 12–18 plants per genotype were used for measuring the mature seed yield-related traits. Florets were marked at the time of fertilization to track developing seeds. 1 DAF, plants were kept in either control conditions (16 h light and 8 h dark at 28 ± 1 °C and 23 ±  °C) or moved to a greenhouse setup for a moderate HS treatment (16 h light and 8 h dark at 36 ± 1 °C and 32 ± 1 °C). The plants were subjected to HS treatment for either 2–4 DAF (HS-I), or 2–10 DAF (HS-II). Afterward, plants were moved back to control temperature conditions and harvested at physiological maturity to analyze mature grain yield-related parameters (Additional file 1b).

### Design of customized chamber

LI-6800 compatible customized cylindrical chamber (length: 25.4 cm; radius: 2.8 cm) was designed by Li-Cor Inc., Lincoln, NE). This chamber is compatible for mounting on the standard sensor head and the chamber dimensions were determined based on the ability to air pump to circulate air using the standard equipment (Additional file 3). The design of the customized chamber allows it to take the advantage of built-in mixing fan of Licor-6800 device to mix the chamber air. Once the chamber is mounted on to the sensor head, LI-6800 recognizes it and provides the option to measure area-based fluxes. However, for the accurate estimation of gas exchange at per unit level area should be determined independently, and we holistically measured it at 3D level by using PI-Plat (as discussed above). An additional quantum sensor was installed on the customized chamber to measure panicle-level incident light. Exhaust ports of the sensor head remained unaffected while mounting the chamber. Thus, chamber installation did not hinder the control of air temperature while taking measurements (Additional file 10). The cylindrical chamber is made up of transparent material to avoid shading and is open at one end to facilitate the insertion of an inflorescence organ (rice panicle in this study). After inserting the panicle into the chamber, we closed the open end of the chamber with a slit rubber stopper without damaging the panicle stalk. The length of the cylinder was adequate to freely accommodate a rice panicle at a time. To prevent the air leakage from the chamber, we further sealed the rubber stopper with modeling clay each time after inserting the panicle.

### Verification of the customized chamber functioning

To verify the functioning of the customized chamber we measured the leaf level gas exchange parameters using the customized chamber, by following a similar approach as that of the panicle (discussed above). The observed leaf-based customized chamber readings were then compared with readings obtained from a traditional fluorometer (using the same leaf) (Additional file 8). We observed that values for photosynthetic parameters obtained from both chambers were similar (Additional file 8). The customized chamber has been designed for carefully accommodate a stalk bearing inflorescence organs and allows complete control of the leakage. However, insertion of leaf into the customized chamber causes minimal leakage due to its morphological features.

### Leaf and panicle photosynthetic measurements

Two LI-6800 (LI-COR) devices were used in parallel to measure leaf and panicle-based gas exchange variables (Fig. 4). For panicle-based gas exchange measurements, a customized chamber was mounted on to the sensor head of one of the LI-6800 devices (details are discussed below). All the measurements were recorded at two time points i.e., 4 and 10 days after fertilization using the plants growing separately under control (16 h light and 8 h dark at 28 ± 1 °C and 23 ±  °C) and HS (16 h light and 8 h dark at 36 ± 1 °C and 32 ± 1 °C) conditions. All photosynthetic measurements were recorded between 1100–1400 h. For photosynthetic measurements, the environmental conditions were set as: Relative humidity chamber at 50%, flow rate at 700 µmol s^−1^, chamber pressure at 0.05 kPa, light intensity at 800 µmol m^−2^ s^−1^, and reference CO_2_ at 400 µmol mol^−1^. LI-6800 warm-up tests were conducted every time before the actual measurements to control the error rates. Air leakage was effectively controlled for both the LI-6800 devices measuring leaf-based and panicle-based gas exchange measurements using rubber gaskets and modeling clay, respectively.

To maintain the incident radiation intensity between 800–900 µmol m^−2^ s^−1^ in the greenhouse setting, two adjustable additional LED lights (Vipar Spectra; Model: V300) were used as a source of diffused light. The LED lights served as a diffused light source specifically for the panicle measurements. These LED lights included IR (Infrared) LEDs that looked dim/invisible and operated at input voltage 120 V and 60 Hz frequency. Plants were first acclimatized to the artificial light source for 15–20 min before recording the photosynthetic measurements. Following the acclimatization, panicles were carefully inserted into the cylindrical chamber. Once the device started recording the gas exchange readings, we waited for reading stabilization before logging the values.

The parameters considered for photosynthetic measurements were *A*_*leaf*_ (leaf carbon assimilation), *gsw*_*leaf*_ (leaf stomatal conductance), *E*_*leaf*_ (apparent leaf transpiration rate), *VPD*_*leaf*_ (leaf to air vapor pressure deficit), *A*_*panicle*_ (panicle carbon assimilation), and *E*_*panicle*_ (apparent panicle transpiration rate). The term “apparent” transpiration rate was used in this study to distinguish it from the transpiration rate occurring under natural unenclosed conditions. Furthermore, we calculated water use efficiency (WUE) of leaf (*WUE*_*leaf*_) and panicle (*WUE*_*panicle*_) separately by dividing respective carbon assimilation rate (*A*) with their apparent transpiration rate (*E*).

### Panicle imaging and downstream analysis

#### Image acquisition

We utilized the Panicle Imaging Platform (PI-Plat) to capture rice panicle images [24, 25, 46]. Briefly, PI-Plat is comprised of a customized wooden chamber (Additional file 2) with a circular wooden board, parallel to the floor, having an aperture at its center. To reduce the interference of light and enhance the image segmentation quality during image processing, the inside of the wooden chamber was painted black. Plants marked for imaging were brought into the chamber, and the primary panicle of the plant was passed through the aperture. The primary panicle was clung to a threaded metal hook attached to the top of the circular wooden chamber, ensuring the panicle stabilization. A rotary apparatus hosting two Sony α6500 cameras and LED lights (ESDDI PLV-380, 15 watts, 500 LM, 5600 K) rotated 360° around the panicle. With the built-in time-lapse application, each camera took an image per second for 1 min. The two cameras generate 120 images for one panicle with a resolution of 6000 × 4000 pixels. The Sony a6500 cameras with following adjustable parameters for the camera while image acquisition: ISO value at 1600, shutter speed at 1/30 s, and aperture value at f/22. Color checkerboards were placed on the chamber and table to facilitate camera parameters recovery and correspondence detection in paired images [55].

### 3D point cloud reconstruction

Captured panicle images were pre-processed to remove the background. To achieve this, images were first converted from the red, green, and blue (RGB) color space into the hue, saturation, and value (HSV) color space. Then, we implemented color thresholding using the MATLAB application “colorthresholder”. Numerous estimation tests using the MATLAB application “colorthresholder” demonstrated that if the hue, saturation, and value were controlled in the ranges of 0–1, 0–1, and 0.15–1, respectively, the background can be effectively removed. Therefore, the pixels were removed if their corresponding hue, saturation, and value were not in the range of 0–1, 0–1, and 0.15–1, respectively. Following the background removal using color thresholds, the residues of the noise (outlines of the black wooden board and chamber) were removed by denoising the pre-processed images. The percentage of incorrect removal of the points that probably belong to the panicle is very low (0.1%), as per our assessment. Therefore, the image pre-processing and background removal should have limited effects on the panicle 3D point cloud generation. Next, the pre-processed images were used to reconstruct 3D point clouds for each panicle. To reconstruct the Panicle's point cloud, we implemented the Multi-View Environment (MVE) pipeline [55]. The MVE pipeline detected and matched the image features in the pre-processed images. A parse point cloud was generated based on matched image features. The parameters of cameras, including position and orientation, were also extracted in this process. Afterward, a dense point cloud was generated by calculating the depth information for each pixel in each image using the cameras' parameters. Finally, floating scale surface reconstruction (FSSR) [56] was implemented to denoise the dense point cloud.

The reconstructed point clouds of the MVE pipeline included all the objects in the scene. We removed uninteresting objects from the original point cloud by implementing color thresholding and connected component labeling to calculate the panicle features in the next section. First, we segmented the panicle's point cloud cluster by computing the Visible Atmospherically Resistant Index (VARI) [57] for each point in the point cloud. The formula in the Eq. (1) is used to decide whether the segmented point cloud is the panicle or uninterested background stuff. The MVE pipeline calculates not only the position of points in the constructed point cloud but also their color based on the images. The color of each point is presented as intensity in R/G/B channel. Using the intensity, we calculate VARI to decide whether a cluster is a panicle. Equation (1) shows the formula of VARI, where R, G, and B mean the corresponding intensity of a point in the RGB color space.

1 $$VARI=\frac{G-R}{G+R-B}$$

The cluster containing the maximum number of points whose VARI > 0.1 is considered as the panicle. Then, we filtered out uninteresting points in the cluster, for instance, plant labels. A representative image of the final point cloud that includes only the panicle is shown in Fig. 1a.

### Trait extraction

In this study, each point cloud was voxelized for volume quantification [58]. The corresponding resulting volume was then used to extract traits of interest, for instance, voxel count and color intensity [46]. Also, we calculated the projected surface area. The projected surface area was used to estimate the surface area of the panicle. We first calculated point cloud's main directions using principal component analysis (PCA) to compute the projected surface area. There were three main directions in a given 3D point cloud. We built 3D coordinate system using the first main direction as Z-axis and the other two directions as the X- and Y-axes. The origin of the system was defined as the lowest point of the point cloud, which was located at the bottom of the panicle. Then, we generated a plane using Y-axis as the norm. After projecting the point cloud of the panicle onto the plane, we calculated the projected surface area as the area of the region enclosed by the boundary of the projected 2D points (Fig. 1b). Afterward, we rotated the plane around the Z-axis and calculated the projected surface area every 5 degrees. In total, we captured 36 projected surface areas. We finally extracted the maximal projected area, the minimal projected area, and the averaged projected area from these results. We used averaged projected area for the final analysis and normalization of panicle’s photosynthetic parameters. We also computed the projection area when the plane was perpendicular to the X-axis and Y-axis. Apart from computing the image-derived traits (projected panicle area, voxel counts, and color intensity) from an entire panicle, we also examined additional traits extracted from local regions of the panicles. We divided the 3D panicle into 10 equal sections along the Z-axis to generate 10 slices. For each slice, we analyzed the corresponding traits (i.e., point count and point color). The analysis of sliced 3D traits enabled us to examine spatial and temporal variation in the development of grains on a particular panicle.

### Correlation analysis

We considered data from 3 digital (green pixels proportion, voxel count, and projected panicle area) and four physiological (A_*panicle*_, E_*panicle*_, A_*leaf*_, E_*leaf*_) traits for computing a pairwise Pearson correlation (PCC). Each trait consisted of an observation from three biological replicates under control and HS from accessions TEJ-1 and TEJ-2. PCC between a pair of traits was computed in RStudio v.1.2.5033 platform. We computed PCC separately for TEJ-1 and TEJ-2 at 10 DAF under HS, as the two accessions had a contrasting performance at this time point under HS. The correlation matrix plot and the significance level was generated using the ‘chart.Correlation’ function incorporated in the 'PerformanceAnalytics' package.

### Mature seed analysis

To assess the effect of moderate HS on mature seeds, we first evaluated only florets marked at the time of fertilization [59]. For this, we scored the total number of fully developed and unfilled or completely sterile seeds to calculate percentage fertility by using the formula:

$${Percent\, fertility}=\frac{{No\, of\, fully\, developed\, seeds}}{{No\, of\, fully\, developed\, seeds+completely\, sterile\, seeds}}  \times 100$$

The dehusked mature seeds were used to measure (i) morphometric parameters (length, width), (ii) single grain weight, (iii) percent fertility. Morphometric analysis was performed on 350–1000 marked seeds from 20–40 plants using *SeedExtractor* [47]*.* Secondly, to have insights into yield-related parameters at a whole plant level, we evaluated all the seeds for percentage fertility and total seed weight per plant.

## Supplementary Information

**Additional file 1. **(a) Experimental plan for panicle imaging and gas exchange measurements. Triangles represent the time points where imaging was done, and photosynthesis parameters were measured. (b) Experimental plan for taking mature seed physiology measurements (details described in methodologies).

**Additional file 2. **Image acquisition setup using PI-Plat imaging platform.

**Additional file 3. **Setup of the customized chamber used for taking panicle gas exchange measurements (with LICOR-6800#1). The inset picture shows in detail the dimensions of the transparent chamber used in this study.

**Additional file 4. **(a)Stomatal conductance (gsw) and (b) vapor pressure deficit (VPD) of flag leaf of TEJ-1 and TEJ-2 developing under control and heat stress conditions at 4 and 10 DAF. For statistics, student’s t-test was conducted separately for each genotype to compare each temperature treatment between the time points. Significant differences are indicated by different letters. Error bars represent ±SE.

**Additional file 5. **Percent change in A*leaf *and A*panicle *at 10 DAF under HS as compared to respective control values in TEJ-1 and TEJ-2. Error bars represent ±SE.

**Additional file 6. **Quantification of single grain weight (mg), spikelet fertility (%), grain length (mm), and grain width (mm) from marked seeds evaluated at the time of physiological maturity.

**Additional file 7. **Shift in voxel count resolved into 3D slices using the panicle point cloud (a) TEJ-1 and (b) TEJ-2.

**Additional file 8. **Measurements of A_leaf_ and E_leaf_ from randomly selected young green leaf (not flag leaf) of TEJ-1 and TEJ-2 plants using sensor head equipped with traditional leaf chamber (light blue) and customized cylinder (dark blue) under control temperature conditions.

**Additional file 9. **Water use efficiency measurements for (a) leaf (WUE_leaf_) and (b) panicle (WUE_panicle_) under control and HS for TEJ-1 and TEJ-2. For statistics, student’s t-test was conducted separately for each genotype to compare each temperature treatment between the time points. Significant differences are indicated by different letters. Error bars represent ±SE.

**Additional file 10. **Air temperature measurements obtained from LI-COR 6800 for leaf (Air temp leaf) and panicle (Air temp panicle) under control and HS for TEJ-1 and TEJ-2. For statistics, student’s t-test was conducted separately for each genotype to compare each temperature treatment between the time points. Significant differences are indicated by different letters. Error bars represent ±SE.

## Data Availability

The raw datasets generated and used in this study are available from the corresponding author on reasonable request.

## Abbreviations

HS: Heat stress
DAF: Days after fertilization
PPA: Projected panicle area
VC: Voxel count
R/G: Red/Green pixels
3D: Three dimensional
VPD: Vapor pressure deficit
