# Supplementary figures and images for: A non-destructive approach for measuring rice panicle-level photosynthetic responses using 3D-image reconstruction
Source: Plant Methods. 2022 Nov 28;18:126. doi: 10.1186/s13007-022-00959-y (PMC9703705; doi:10.1186/s13007-022-00959-y)

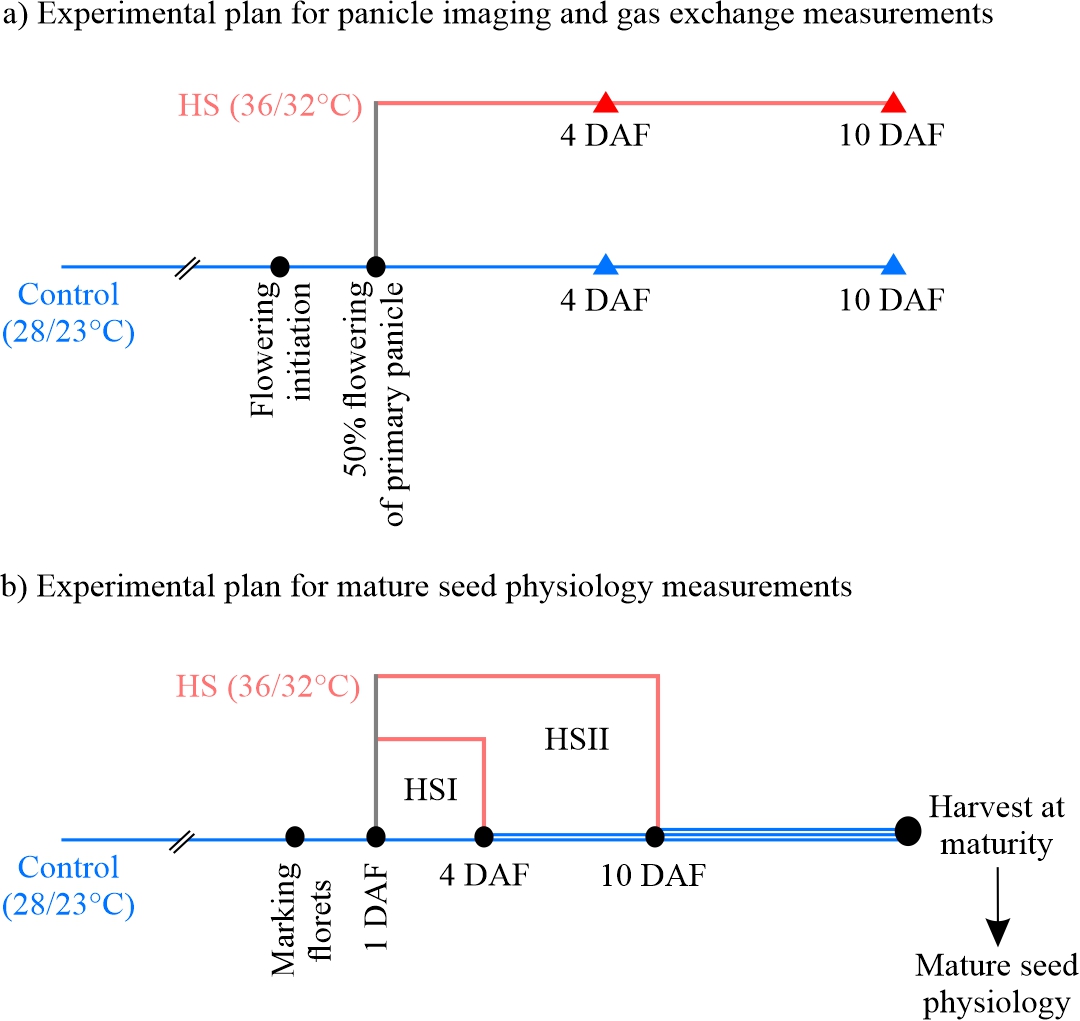

Supplement: Supplementary file 1 — Additional file 1. (a) Experimental plan for panicle imaging and gas exchange measurements. Triangles represent the time points where imaging was done, and photosynthesis parameters were measured. (b) Experimental plan for taking mature seed physiology measurements (details described in methodologies). [file 13007_2022_959_MOESM1_ESM.jpg]

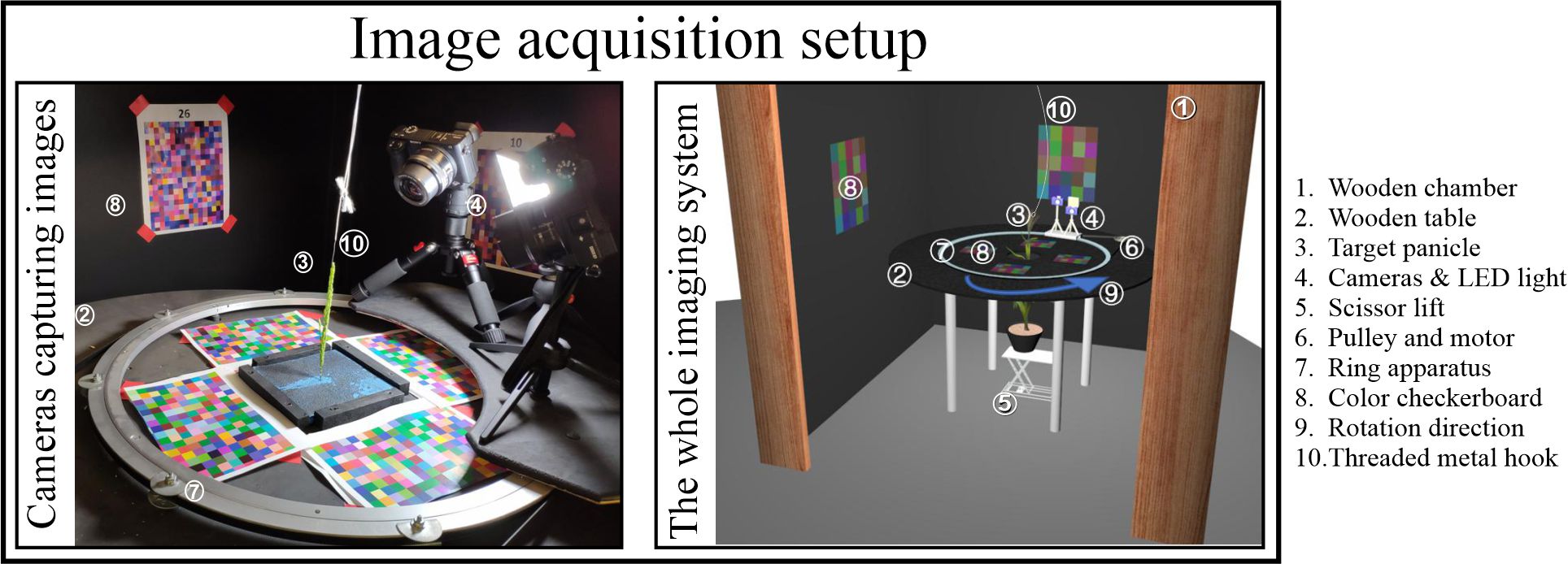

Supplement: Supplementary file 2 — Additional file 2. Image acquisition setup using PI-Plat imaging platform. [file 13007_2022_959_MOESM2_ESM.jpg]

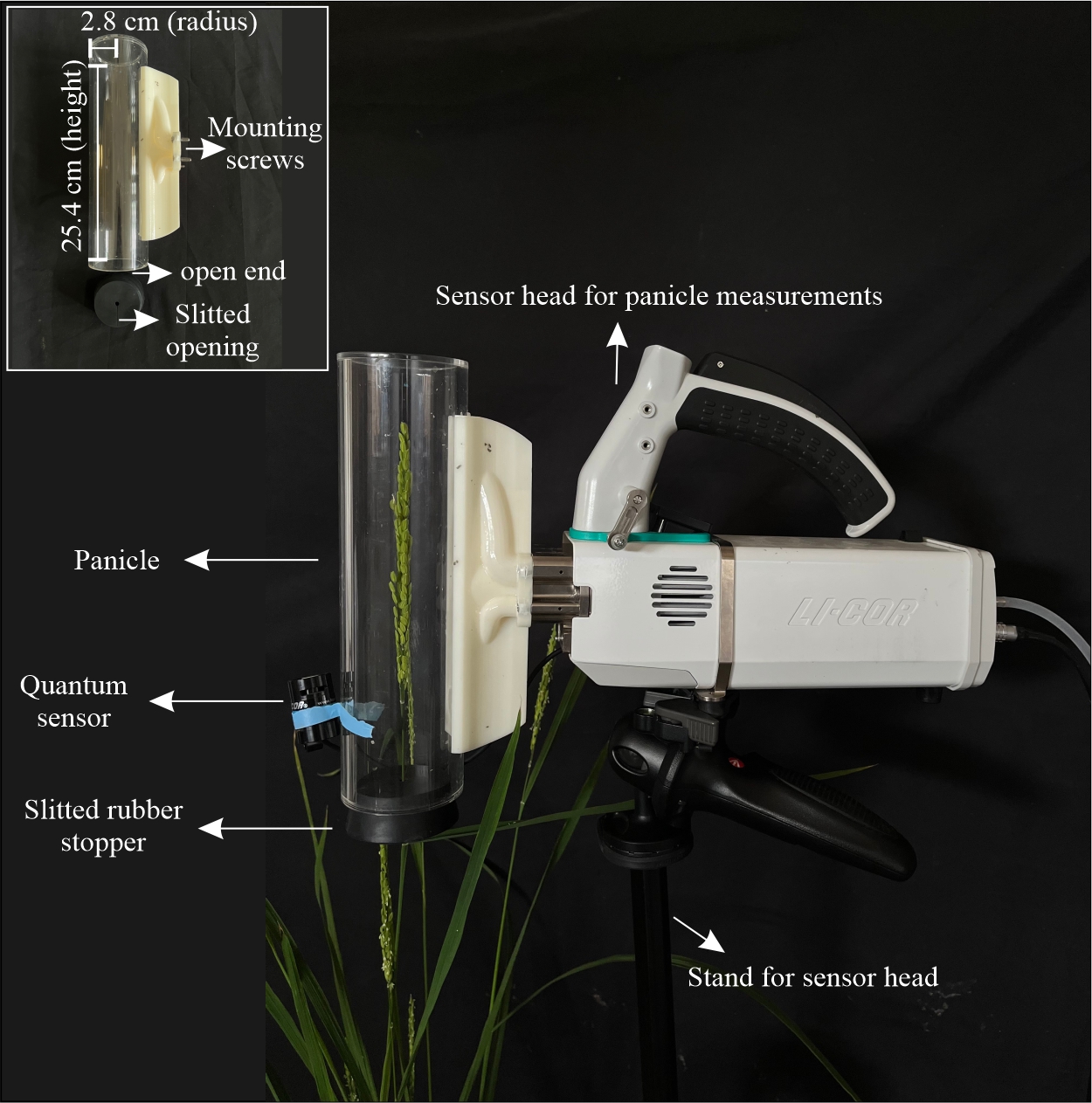

Supplement: Supplementary file 3 — Additional file 3. Setup of the customized chamber used for taking panicle gas exchange measurements (with LICOR-6800#1). The inset picture shows in detail the dimensions of the transparent chamber used in this study. [file 13007_2022_959_MOESM3_ESM.jpg]

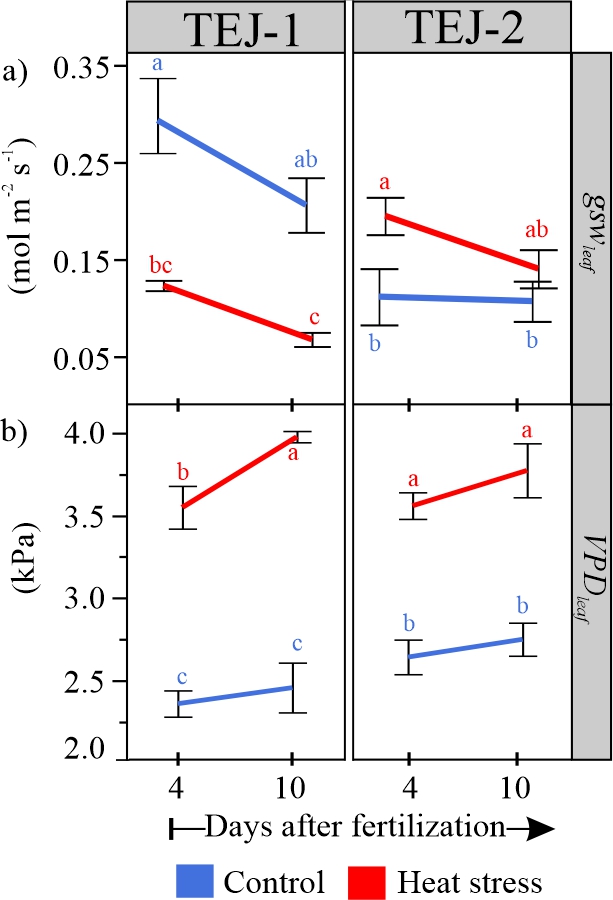

Supplement: Supplementary file 4 — Additional file 4. (a)Stomatal conductance (gsw) and (b) vapor pressure deficit (VPD) of flag leaf of TEJ-1 and TEJ-2 developing under control and heat stress conditions at 4 and 10 DAF. For statistics, student’s t-test was conducted separately for each genotype to compare each temperature treatment between the time points. Significant differences are indicated by different letters. Error bars represent ±SE. [file 13007_2022_959_MOESM4_ESM.jpg]

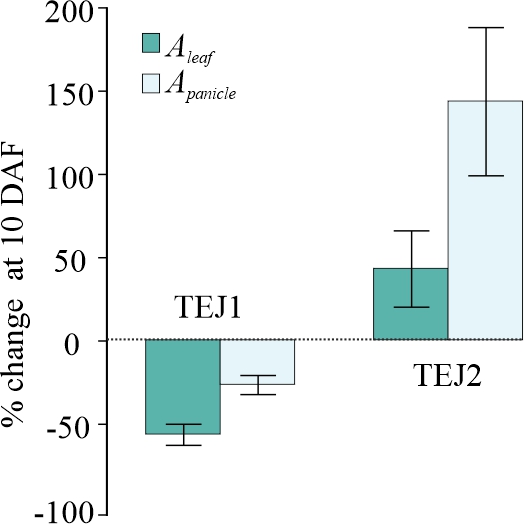

Supplement: Supplementary file 5 — Additional file 5. Percent change in Aleaf and Apanicle at 10 DAF under HS as compared to respective control values in TEJ-1 and TEJ-2. Error bars represent ±SE. [file 13007_2022_959_MOESM5_ESM.jpg]

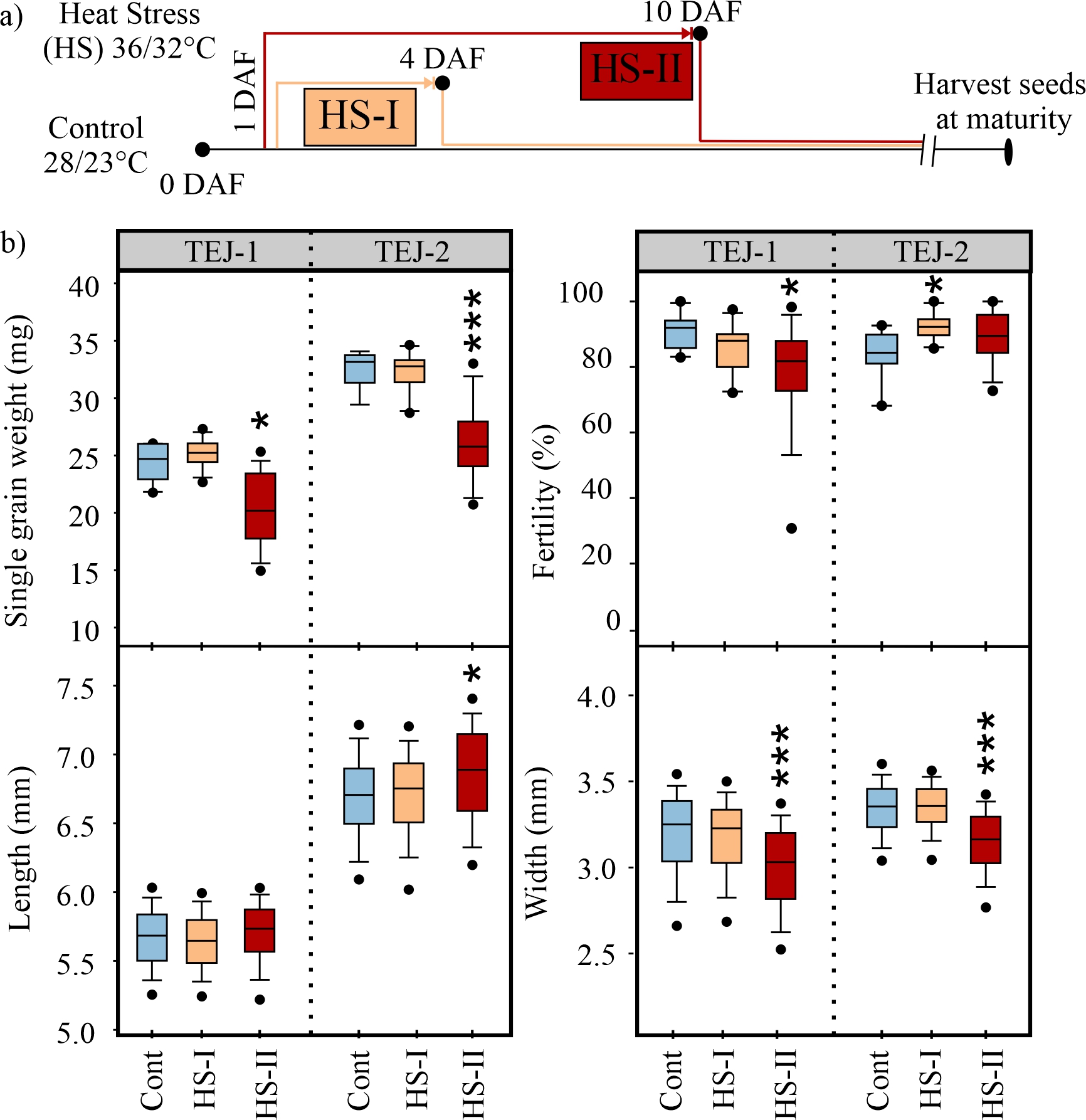

Supplement: Supplementary file 6 — Additional file 6. Quantification of single grain weight (mg), spikelet fertility (%), grain length (mm), and grain width (mm) from marked seeds evaluated at the time of physiological maturity. [file 13007_2022_959_MOESM6_ESM.jpg]

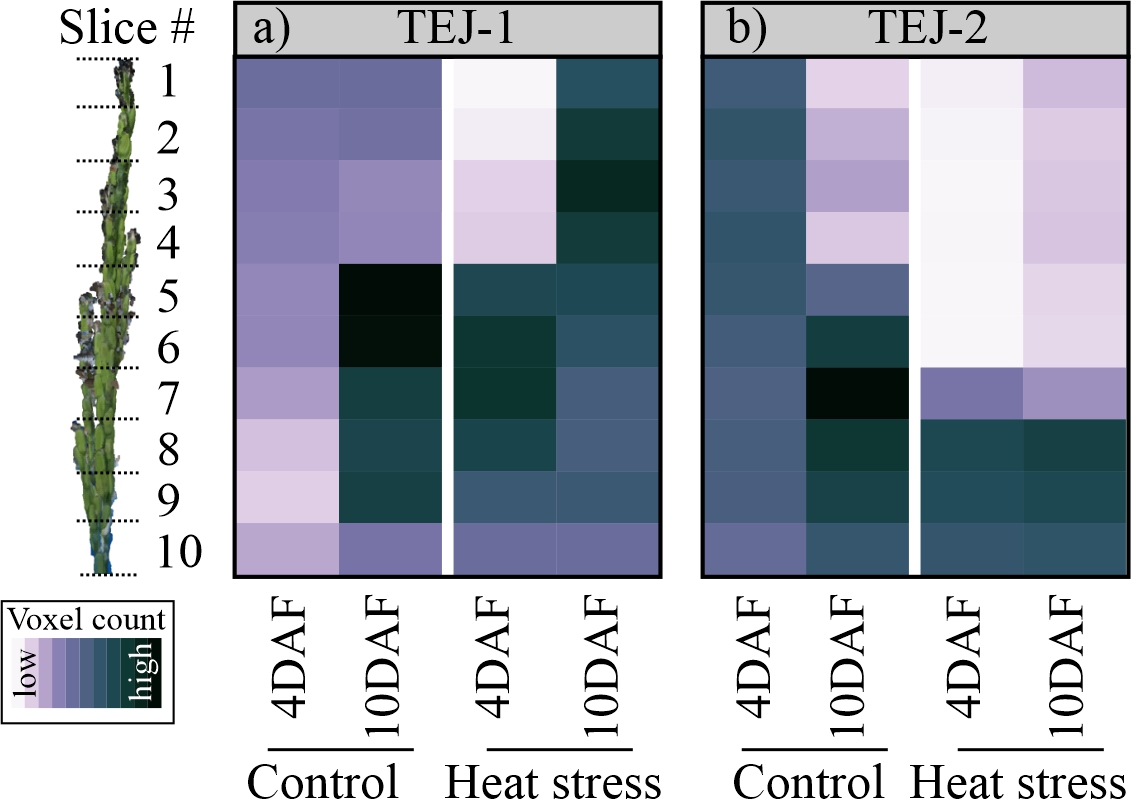

Supplement: Supplementary file 7 — Additional file 7. Shift in voxel count resolved into 3D slices using the panicle point cloud (a) TEJ-1 and (b) TEJ-2. [file 13007_2022_959_MOESM7_ESM.jpg]

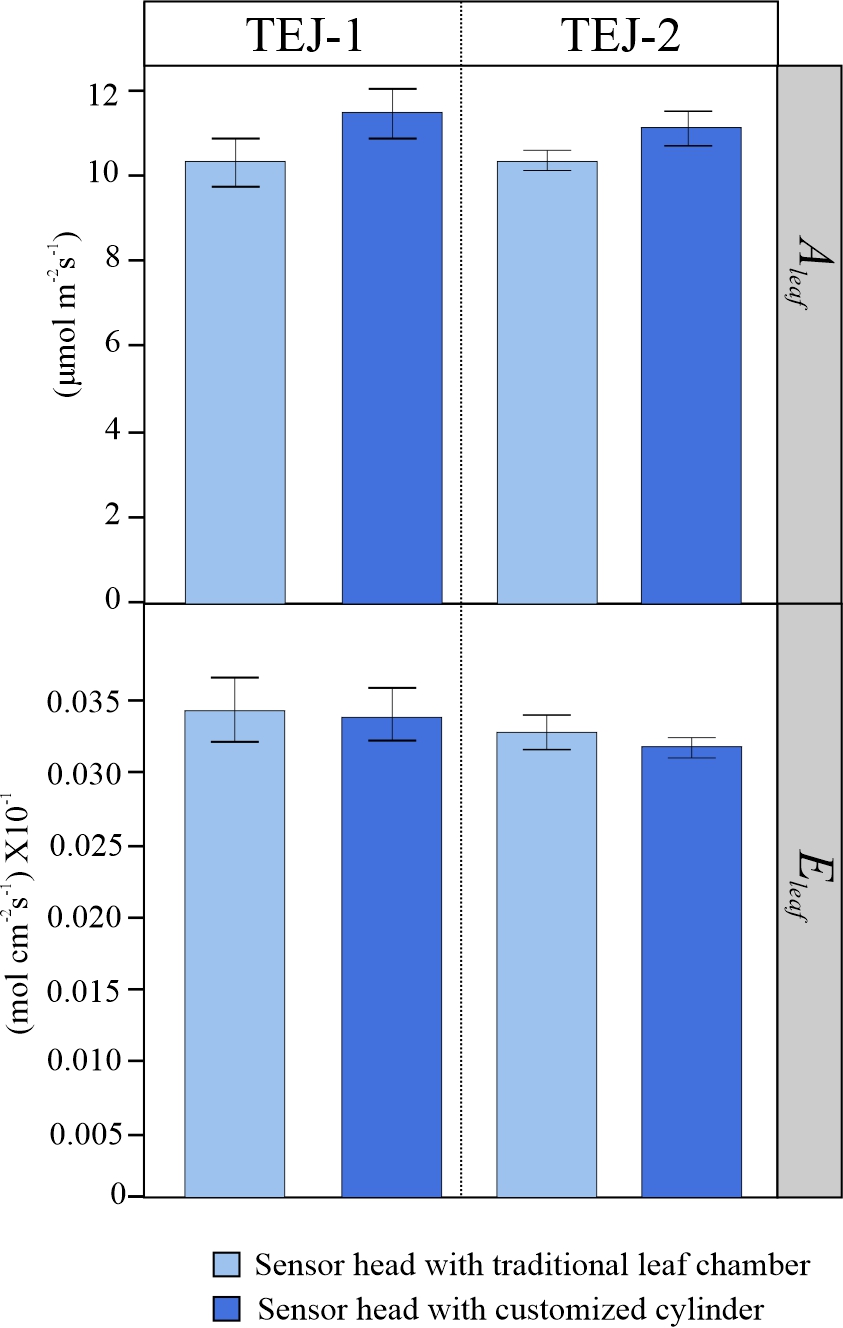

Supplement: Supplementary file 8 — Additional file 8. Measurements of Aleaf and Eleaf from randomly selected young green leaf (not flag leaf) of TEJ-1 and TEJ-2 plants using sensor head equipped with traditional leaf chamber (light blue) and customized cylinder (dark blue) under control temperature conditions. [file 13007_2022_959_MOESM8_ESM.jpg]

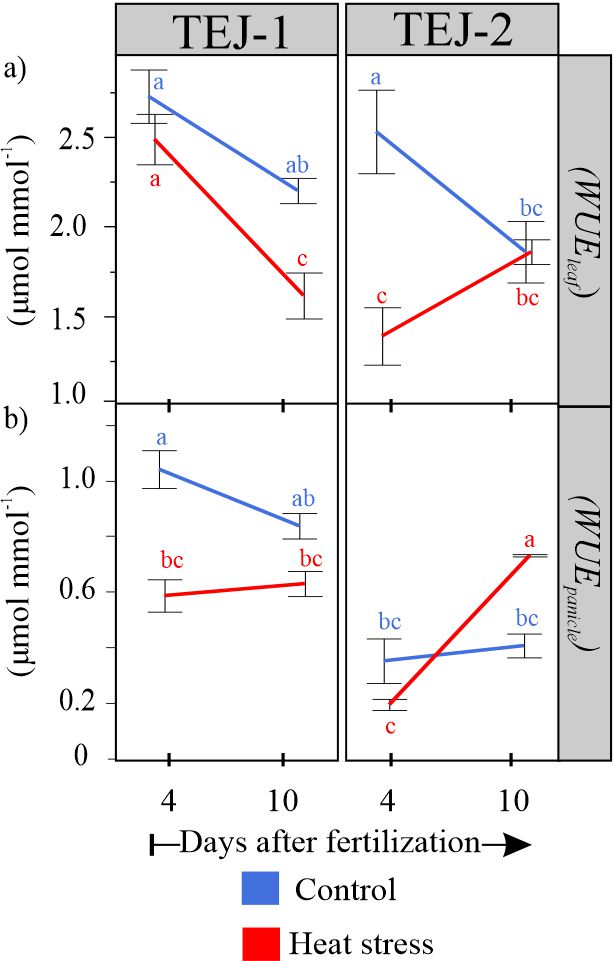

Supplement: Supplementary file 9 — Additional file 9. Water use efficiency measurements for (a) leaf (WUEleaf) and (b) panicle (WUEpanicle) under control and HS for TEJ-1 and TEJ-2. For statistics, student’s t-test was conducted separately for each genotype to compare each temperature treatment between the time points. Significant differences are indicated by different letters. Error bars represent ±SE. [file 13007_2022_959_MOESM9_ESM.jpg]

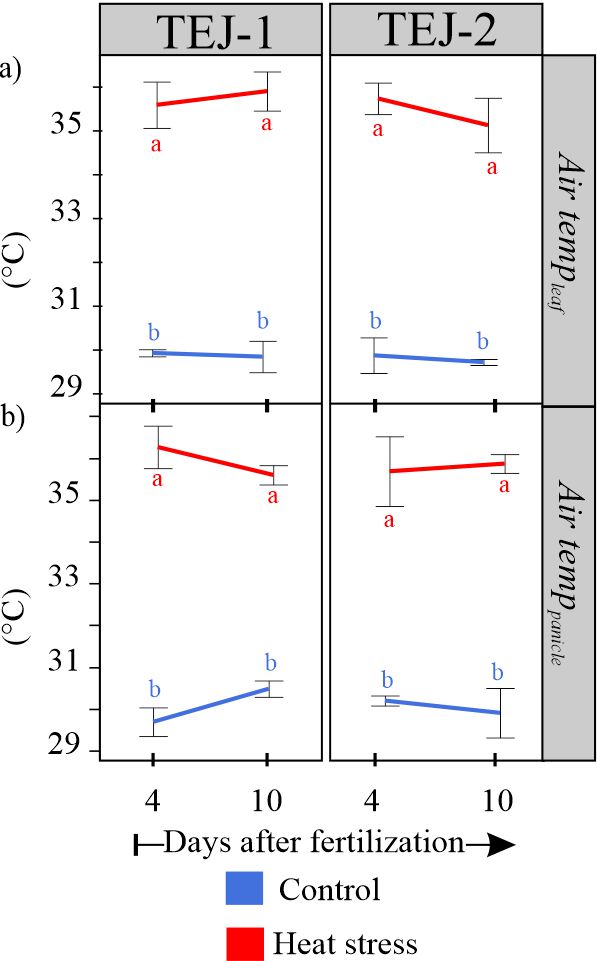

Supplement: Supplementary file 10 — Additional file 10. Air temperature measurements obtained from LI-COR 6800 for leaf (Air temp leaf) and panicle (Air temp panicle) under control and HS for TEJ-1 and TEJ-2. For statistics, student’s t-test was conducted separately for each genotype to compare each temperature treatment between the time points. Significant differences are indicated by different letters. Error bars represent ±SE. [file 13007_2022_959_MOESM10_ESM.jpg]
